# An Associated Representation Method for Defining Agricultural Cases in a Case-Based Reasoning System for Fast Case Retrieval

**DOI:** 10.3390/s19235118

**Published:** 2019-11-22

**Authors:** Zhaoyu Zhai, José-Fernán Martínez Ortega, Victoria Beltran, Néstor Lucas Martínez

**Affiliations:** Departamento de Ingeniería Telemática y Electrónica (DTE), Escuela Técnica Superior de Ingeniería y Sistemas de Telecomunicación (ETSIST), Universidad Politécnica de Madrid (UPM), C/Nikola Tesla, s/n, 28031 Madrid, Spain; jf.martinez@upm.es (J.-F.M.O.); mv.beltran@upm.es (V.B.); nestor.lucas@upm.es (N.L.M.)

**Keywords:** association relation, case representation, case-based reasoning, knowledge management, smart agriculture

## Abstract

As an artificial intelligence technique, case-based reasoning has considerable potential to build intelligent systems for smart agriculture, providing farmers with advice about farming operation management. A proper case representation method plays a crucial role in case-based reasoning systems. Some methods like textual, attribute-value pair, and ontological representations have been well explored by researchers. However, these methods may lead to inefficient case retrieval when a large volume of data is stored in the case base. Thus, an associated representation method is proposed in this paper for fast case retrieval. Each case is interconnected with several similar and dissimilar ones. Once a new case is reported, its features are compared with historical data by similarity measurements for identifying a relative similar past case. The similarity of associated cases is measured preferentially, instead of comparing all the cases in the case base. Experiments on case retrieval were performed between the associated case representation and traditional methods, following two criteria: the number of visited cases and retrieval accuracy. The result demonstrates that our proposal enables fast case retrieval with promising accuracy by visiting fewer past cases. In conclusion, the associated case representation method outperforms traditional methods in the aspect of retrieval efficiency.

## 1. Introduction

As an important technique in artificial intelligence, case-based reasoning (CBR) is defined as the process of reusing experiences to deal with current situations that are similar to the ones that have been solved and stored beforehand [1]. It mimics how humans would perform reasoning and learning. As a more psychologically plausible model of human reasoning, the case-based reasoning technique has been widely employed for building intelligent systems in various fields like healthcare [2,3,4], fault diagnosis [5,6,7], emergency response [8,9,10], and agricultural decision-making [11,12,13].

In smart agriculture, various sensors are deployed for collecting data. One of the challenges for processing these data is to transfer the sensor measurements into practical knowledge [13]. CBR is acknowledged as a powerful technique for building intelligent agricultural systems to support knowledge management where metadata descriptions are used for characterizations [14]. These characterizations can be presented by cases, consisting of problem descriptions and corresponding solutions. Thus, the intelligent agricultural systems can benefit from combining the case-based reasoning technique and sensing technologies.

Since CBR has promising advantages like ease of use and precise response [15], smart agriculture has adopted this technique for building intelligent systems, aiming at providing farmers with quick decision supports for farming operation management by referring to past experiences. Applying CBR to smart agriculture has the following merits. On the one hand, learning from successful experiences enables us to improve the decision-making capability and efficiency. On the other hand, prediction models can be developed by recognizing the patterns within cases.

**Figure 1:**
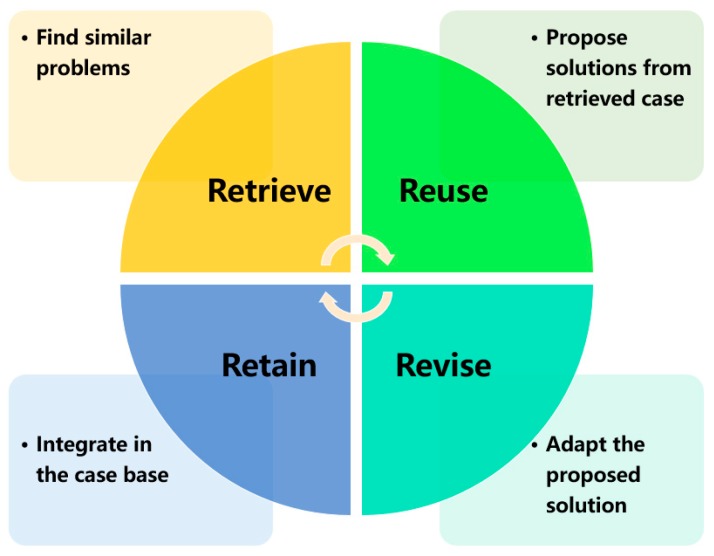
A generic workflow of case-based reasoning.

CBR is usually formalized by a well-recognized 4R circle, including retrieve, reuse, revise, and retain [16]. The workflow of CBR is presented in Figure 1.

In Figure 1, the retrieval process firstly compares the new case with historical ones for matching the most similar past case. The solution for this retrieved case is reused as guidance for resolving the current situation. A revision process is required to update the proposed solution in case current and past situations have major differences. Then the updated solution is employed and the solved case is stored in the case base as past experiences. Apart from the 4R circle, few researchers explored the aspect of case representation when applying CBR. In most situations, it is assumed that the case base has been already available for the first process (Retrieve). However, case representation is an essential factor in CBR because the reasoning capability of CBR systems mainly depends on the structure and content of cases. Thus, a 5R circle is expected, including the step of case representation [17].

Experiences in the CBR systems are stored by the form of cases, thus the representational formalism plays a significant role in CBR [18]. Case representation is the task of enabling CBR systems to recognize, store, and process past contextualized experiences [19]. Cases can be represented by simple feature vectors [20], or they can be represented by using any representational formalisms like the following.

- Frame-based representation [21]: A frame organizes knowledge in slots that describe various attributes and characteristics of the object. It is a natural way for the structured and concise representation of knowledge.
- Object-oriented representation [22]: The object-oriented representation is a common way of defining IS-A, HAS-A, and PART-OF relationships. Cases are represented by a collection of object classes.
- Predicate-based representation [23]: A predicate is a relation among objects, consisting of a condition part (IF) and an action part (THEN). This representational formalism is usually found in literature about fuzzy logic, neural network, and rough set theory.
- Semantic nets [24]: This representation method involves nodes and arcs that link nodes. Each node either represents an object or a concept and an arc represents the relation between nodes.
- Rules representation [25]: The rule-based representation usually adopts rules like IF-THEN and it is applicable in a relative simple domain.

The choice of a particular representational formalism is largely determined by the information to be stored within a case. However, most of current case representational formalisms fail to address the interrelation between cases. In the case base, each case is treated individually. For retrieving similar past cases, the retrieval algorithms have to compare all the cases sequentially, leading to low retrieval efficiency when a large volume of cases is stored in the case base. Low retrieval efficiency usually results in poor performance of case-based reasoning systems.

In this paper, an associated case representation method is proposed, enabling fast case retrieval in agricultural CBR systems. Typically, a case consists of two parts, including problem descriptions and solution explanations. On this foundation, an additional part is extended in this typical case structure, named association part. As a consequence, our proposal follows a “problem–solution-association” structure. Each case in the case base is interconnected with several associated ones, which have great commonalities or differences. Once a new agricultural case is reported, its features are compared with historical data by similarity measurements for identifying a relative similar past case. Then, comparisons between the new case and associated ones are conducted preferentially until the most similar past case is matched. Due to this association relation, the most similar past case can be retrieved by visiting a fewer number of cases. Thus, the efficiency of case retrieval can be significantly improved under the circumstance that a large volume of data is stored in the case base. With such improvements, CBR systems are able to better serve the application domain of smart agriculture.

The rest of this paper is organized as follows. In Section 2, related works on current states of case representation methods are reviewed, including textual representation, the attribute-value pair, and ontology model. In Section 3, the basic content of agricultural cases is analyzed for preparation purpose. The proposed associated case representation is explained in Section 4, including all three parts: problem description, solution explanation, and association relation. Experiments are performed in Section 5, following by comparisons between our proposal and traditional case representation methods in the aspect of retrieval efficiency and accuracy. Lastly, conclusions are drawn in Section 6.

## 2. Related Work

In this section, the current state of case representation methods is reviewed. As a critical factor in CBR systems, case representation decides the reasoning capability and efficiency of case retrieval. Bergmann et al. [19] defined cases as “a contextualized piece of knowledge representing an experience that teaches a lesson fundamental to achieving the goals of the reasoner”. Cases are the foundation of any CBR systems and they can be presented by various forms. Three popular case representation methods, textual representation, attribute-value pair, and the ontology model, are reviewed in this section. The merits and demerits of each representational formalism are summarized. It is worth mentioning that developers and researchers who are interested in adopting the case-based reasoning technique should determine the proper method by themselves according to their requirements (information to be stored) and available resources, as well as the characteristics of case representation methods. To the best of our knowledge, we did not detect any standards for selecting the case representation methods.

### 2.1. Textual Representation

The textual representation method is commonly used in textual case-based reasoning (TCBR) [26]. As a subfield of CBR, TCBR aims at using textual knowledge sources in an automated or semi-automated way for solving problems through case comparisons.

Two research lines have been pointed out by Ozturk and Prasath [27]. The first one focuses on extracting contents of a case from a textual report and populating obtained knowledge into a case base under a structured manner. Bruninghaus and Ashley [28] contributed to this research line by using natural language processing and information extraction methods to automatically extract relevant factual information. The second research line lies on retrieving knowledge from free texts without converting them into structured cases. Dufour-Lussier et al. [29] introduced a method for the automatic acquisition of a rich case representation from free text for process-oriented case-based reasoning.

**Figure 2:**
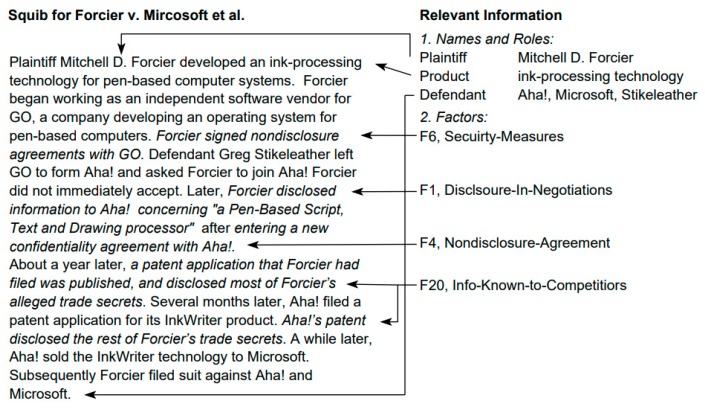
An example of textual representation and its relevant information [28].

It is worth noting that textual cases are usually presented by plain text, resulting in difficulty for systems to understand the information within cases. Thus, this representation method is domain specific and it requires knowledge engineers to determine which features are relevant in the target domain. An example of textual representation is shown in Figure 2.

A major advantage for adopting the textual representation is that it can handle both semi-structured and unstructured documents. This method is sufficient when the amount of information is not too extensive. However, textual representation is domain specific and the retrieval process is complex.

### 2.2. Attribute-Value Pair

**Figure 3:**
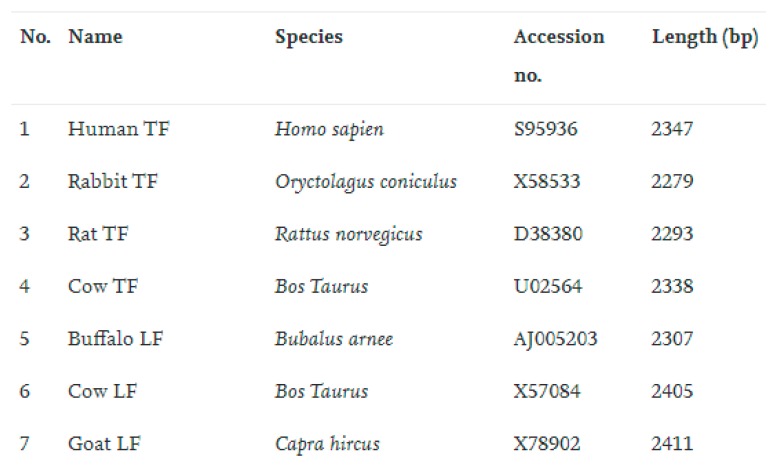
An example of the attribute-value pair [30].

Attribute-value pair, also known as feature vector representation, is the simplest way of defining cases in CBR systems. It is presented as a set of selected features, along with corresponding values [30]. An example of attribute-value pair is presented in Figure 3.

A feature is an individual measurable property or characteristic of an observable phenomenon. It can be defined by numeric attributes, strings, graphs, fuzzy variables, etc. In Figure 3, apart from the case ID, a 4-dimendional feature representation vector is used to reconstruct phylogenetic trees. These features characterize the relative difference of biological sequences. In this example, features “Name”, “Species”, “Accession no.”, and “Length” are key attributes to determine the commonalities between different cases.

Due to ease of use and simplicity, attribute-value pair has been adopted by researchers from all fields. Asadi and Lin [31] employed the feature vector representation in multi-stage document ranking. This representation not only helps the system to save more memory, but also offers greater flexibility. Bringer et al. [32] used the feature vector representation for transforming a fingerprint minutiae template into a binary format with a fixed length. The experimental result demonstrates that their proposal achieves promising improvements for utilization into fast identification algorithms. Svanberg [33] adopted the feature vector representation for identifying similar projects in App Inventor. By measuring the similarity between projects, original projects can be distinguished from unoriginal ones. Yagi et al. [34] proposed an edge-based feature vector representation for a medical image recognition system. The robust nature of the feature vector representation has been verified through the cephalometric landmark identification and expert dentists’ practice.

Concluding from the above literature, it is well acknowledged that attribute-value pair has the merits of simplicity, flexibility, and great robustness. However, there are no relationships or constraints between features in this representation formalism. Due to the lack of domain knowledge, semantic similarity can be hardly measured. Moreover, if a case contains incomplete or ambiguous information, difficulties may be encountered during the process of case retrieval. Thus, improvements of such representation are highly expected.

### 2.3. Ontology Model

Ontology is a formal explicit description of concepts and their relations [35]. It attempts to represent entities, ideas, and events, along with their interdependent properties and relations, on the basis of a system of categories. Within an ontology model, main components include “Individuals” (instances or objects), “Classes” (sets, collections, and concepts of things), “Attributes” (aspects, properties, and features that classes can have), “Relations” (in which way classes and individuals are related to one another), “Rules” (descriptions of logical inferences), “Axioms” (assertions in a logical form), and “Events” (changes of attributes or relations) [36].

**Figure 4:**
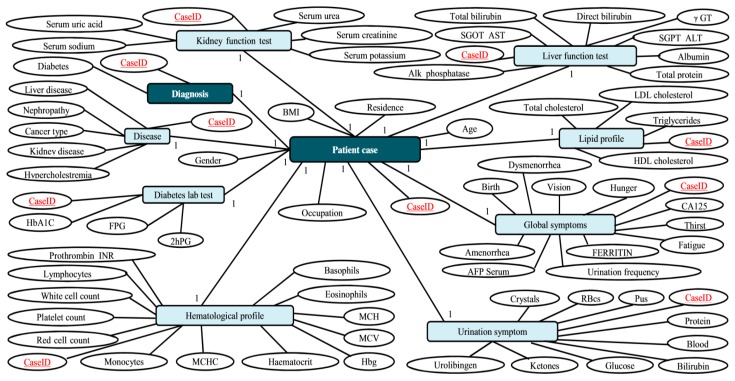
The crisp ontology model used in [38].

Due to its powerful semantic knowledge representation, tremendous contributions have been made by the research community in regards to ontology representations in CBR systems. For instance, Deng et al. [37] employed a semantic vector space model (SVSM) to construct cases based on relevance with semantic fields. The K-nearest neighbor (K-NN) algorithm is used to match the most similar case. El-Sappagh et al. [38] proposed to use ontology for case representation and storage in the domain of diabetes diagnosis. The ontology model used in their proposal is shown in Figure 4. In their proposal, cases are represented as concept instances and their attributes are represented as ontology relations or properties. The values that relation attributes may take are instances of defined within the domain ontology. Bergmann and Schaaf [39] explored the possibility of combining structural case-based reasoning and ontology-based knowledge management. Through their analysis, both techniques show a strong relationship. Meanwhile, the combination of CBR and ontology offers greater flexibility and capability of semantic query.

Though using the ontology representation method improves the semantic performance of CBR systems, this representation requires domain specific knowledge for building the model [40,41,42,43]. Thus, manipulating ontology models is a complex task for ordinary users.

### 2.4. Summary

After reviewing the selected three case representation methods, it is concluded that both the textual representation and attribute-value pair do not concern the interconnection between cases. Normally, each case is treated individually and stored in the case base under a sequential manner. When adopting these two methods, it is inefficient to retrieve similar past cases because all the past cases have to be visited. In regards to the ontology model, though it has the component like “Relations” that links classes and individuals to each other, these associations concern more about upper and lower directories. For example, in [38], a patient case (“Individual”) has global symptoms (“Classes”), while these global symptoms have several “Attributes”, like birth, vision, hunger, etc. From this aspect, it is determined that “Relations” can indicate the internal associations between different components, but not the association between different “Individuals”. As a consequence, all the cases have to be visited as well when retrieving similar past cases, leading to low retrieval efficiency.

Conclusively, one of the potential improvements for the case representation is to mine the relationship between cases, including both similar and dissimilar associations. With such associations, similar past cases might be retrieved efficiently and accurately.

## 3. Basic Content of an Agricultural Case

An agricultural case generally considers two groups of information: environmental data and crop/plant related data [44].

Firstly, environmental data are usually external variables like soil, temperature, humidity, sunlight, wind, pests, diseases, and so on. It plays a significant role in agricultural operation management because it can affect the growth of plants and operation performances. For example, the rising temperature can possibly shorten the growth circle of crops. Agronomists estimate that the growth circle of rice will shorten by more than one week if the temperature increases by one Celsius degree [45]. Another example may refer to deploying unmanned aerial vehicles (UAVs) for farming operations. When the wind speed is over 10 m/s, it is not applicable for launching UAVs [46] due to safety concerns. Conclusively, some key features of environmental data in agricultural cases are summarized in Table 1.

**Figure 5:**
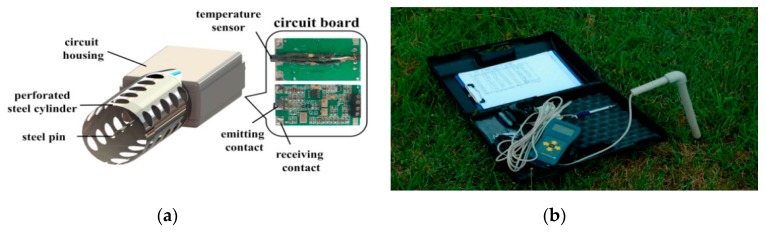
Some latest sensor equipment: (**a**) a perforated cylinder coaxial dielectric sensor for measuring soil water content [47] and (**b**) a time-domain reflectometry sensor for measuring soil moisture [48].

Owing to the advanced sensing technology and Internet of Things, environmental data can be collected and utilized by various sensors deployed in the farmlands. Some latest sensor equipment is shown in Figure 5.

Secondly, crop/plant related data include crop growth, crop yield, stress, plant dry weight, flowering time, root biomass index, planting density, etc. It is necessary to take these variables into account because agricultural decision-making varies in different situations. For instance, the planting density of crops may affect the dilution concentration of insecticides [49]. Intensive planting usually requires a lower dilution. Furthermore, crops are sensitive to the applied insecticides due to their growth stage. Toxic insecticides may be lethal to those crops, which are at the seed stage. Thus, following key features of crop/plant related data are presented in Table 2.

**Figure 6:**
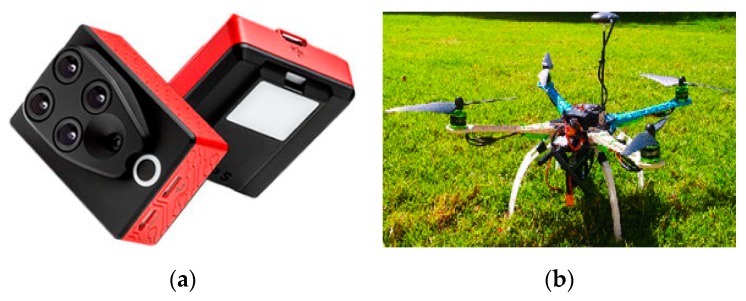
Examples of multispectral sensors for monitoring crop data [50]: (**a**) a multispectral sensor for crop discrimination and (**b**) an airborne multispectral sensor for assessing nitrogen nutrition [51].

For monitoring crops, multispectral sensors have been widely adopted. This type of sensor is usually carried by UAVs or remotely operated drones (RODs). After surveying an area, aerial vehicles can transfer obtained images to processing units for further analyses like feature extraction and pattern recognition. Examples of multispectral sensor applications are shown in Figure 6.

The above two categories of data form the problem description of an agricultural case. In terms of the solution part, it varies in the application domain. For instance, a solution for a pesticide spraying mission should indicate the pesticide type, applied dosage, dilution formula, spraying method, etc. While a solution for assigning tasks to the agricultural machinery should provide the number of vehicles and a task distribution strategy. Since this paper focused on proposing an associated case representation method for fast case retrieval, the solution part was not our concern. Since the process of case retrieval did not evaluate the solution part in this paper, consequently, we were not going to evaluate the solution part.

## 4. Method of Associated Case Representation and Fast Case Retrieval

The proposed associated case representation method is on the basis of the attribute-value pair method. As introduced in Section 2.2, an attribute-value pair consists of selected features and corresponding values. In general, agricultural cases are composed of representative features and corresponding values, like soil moisture, soil temperature, air humidity, sunlight radiation, etc. This is the reason why the attribute-value pair is chosen as the foundation of our proposal. The representative features of agricultural cases are selected from Section 3, including both environmental and crop/plant related data.

### 4.1. Representation Formulism

**Figure 7:**
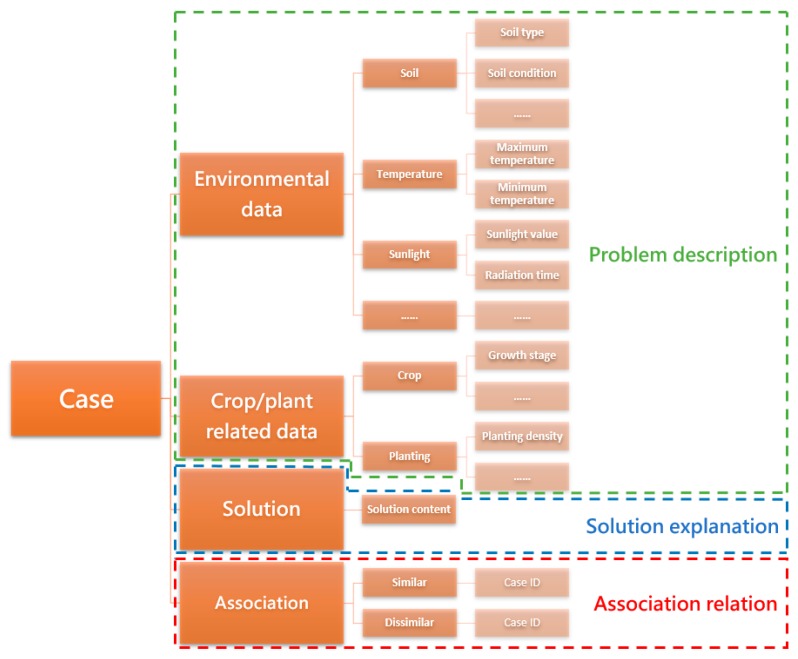
The structure of the associated case representation.

Differing from the typical case representation methods, our proposal extends the “problem–solution” structure with an additional association part. The structure of our proposal is “problem–solution-association”. The association relation enables a single case to interconnect with other relevant cases. This association relation plays a key role in fast case retrieval. Similar cases are interconnected with each other, thus the retrieval process may focus on a smaller range of cases. Figure 7 presents the structure of the associated case representation.

**Figure 8:**
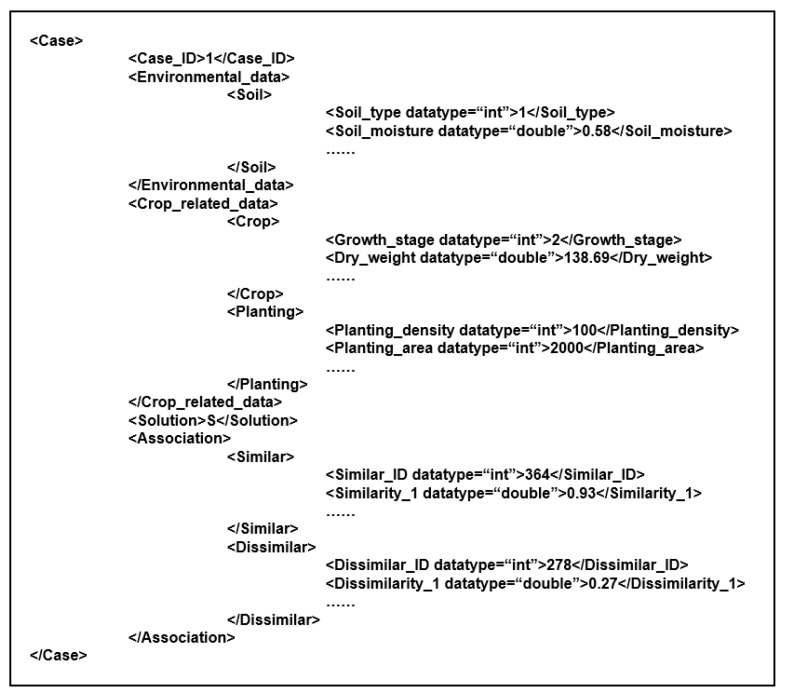
The association case representation in XML.

In Figure 7, the associated case representation follows a “problem–solution-association” structure. For the implementation, we adopted XML to represent and organize the cases in the case base [52], because the structure of XML-based cases is flexible and case storage is relatively independent. Meanwhile, the node custom function in XML enabled us to create new cases conveniently. An example of a XML implementation is presented in Figure 8.

In regards to the association relation between cases, it not only indicates those similar cases, but also dissimilar ones.

- Similar cases: Similar cases are considered to have great commonalities in environmental and crop/plant related data. In our design, each case was associated with three similar ones and stored with case IDs and their corresponding similarity measurements. Once a new case was compared with a past case, the associated similar cases of this past case were compared as well for the reason that the potential most similar case might exist among these associations. Instead of searching the whole case base, the association of similar cases provided the opportunity of measuring the similarity within a smaller range. Consequently, the number of visited cases was reduced, leading to higher case retrieval efficiency.
- Dissimilar cases: Dissimilar cases are considered to have significant differences in environmental and crop/plant related data from the target case. In our design, each case was associated with three dissimilar ones and stored case IDs and their corresponding similarity measurements. The association relation of dissimilar cases was designed to assist the new case in quickly identifying a relative similar case at the very beginning of case retrieval. Instead of searching for cases sequentially, if the similarity between the new case and past case was beyond a given threshold, the comparison with those dissimilar cases would be conducted until a relative similar case was matched.

**Figure 9:**
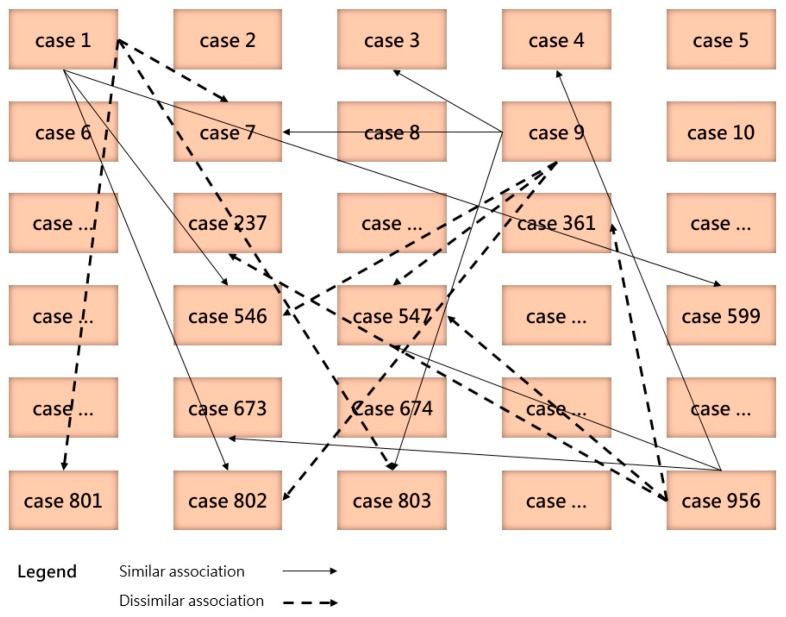
The similar and dissimilar association relations between cases.

It is worth noting that associating with dissimilar cases has a significant distinction to removal of irrelevant cases during case retrieval. For improving the retrieval efficiency, some methods like the rough set theory and filtering techniques were employed. Du et al. [53] adopted the rough set theory for exploring the essential spatial relations. Though the rough set theory is able to reduce the number of cases for similarity measurement by defining the lower and upper approximations, it still has to calculate all cases in the case base for generating a set of qualified cases. Perea-Ortega et al. [54] defined different manual rules to filter each recovered document for the geographic information retrieval task. The rules are designed based on the observation of documents and topics, following a geographic reasoning. A shortcoming of this method is that users have to define filtering rules manually according to their own interests for each retrieval task. Unfortunately, both the rouge set theory and filtering techniques do not build any actual association relations between cases and they are task specific. Thus, our proposal for the associated case representation is promising because it explores the association relations between cases. No matter what the new case is, the efficiency of case retrieval can be guaranteed and no manual work is needed. An example of association relation between cases is presented in Figure 9.

In Figure 9, some parts of the association relations are presented. For example, “case 1” had similar association relations with “case 546”, “case 599”, and “case 802”, while it was considered dissimilar to “case 7”, “case 801”, and “case 803”. After associating cases with each other, the similarity evaluation was limited in a smaller set of cases.

### 4.2. Fast Case Retrieval

**Figure 10:**
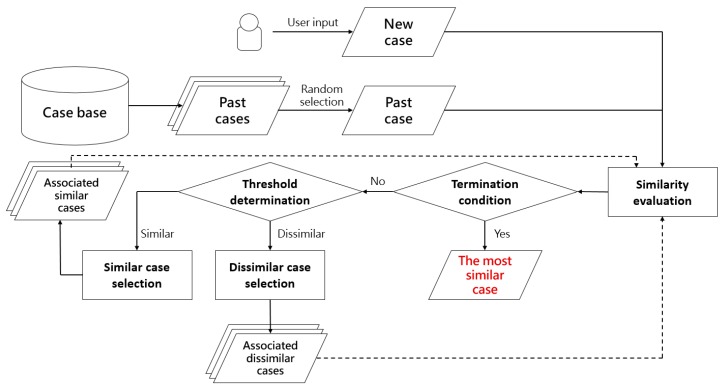
The workflow of fast case retrieval under the proposed associated case representation.

The workflow of fast case retrieval with the proposed associated case representation is presented in Figure 10.

In Figure 10, the proposed associated case representation enabled fast case retrieval by comparing with associated similar and dissimilar cases preferentially, instead of measuring the similarity of all the cases in the case base. The process of similarity evaluation adopts a distance-based measure [55]. Since the problem description of a case is defined by the attribute-value pair, a case can be formulized by a vector. Attributes of vectors are compared with each other and then summed up with weights, as the overall similarity.

For the process of the threshold determination, following rules should be strictly respected.

- Rule 1: Determination of dissimilar cases. When the similarity of compared two cases is less than 50%, the target case (new case) is considered dissimilar to the source case (past case). The source case is assigned with a dissimilar flag.
- Rule 2: Determination of similar cases. When the similarity of compared two cases is greater than 50%, the target case is considered similar to the source case. The source case is assigned with a similar flag.
- Rule 3: Determination of highly similar cases. When the similarity of compared two cases is greater than 75%, the target case is considered highly similar to the source case. An extra flag is assigned to the source case, marking it as highly similar.
- Rule 4: Determination of selecting similar or dissimilar associations for the next iteration. Under the general circumstance, the association with more flags will be chosen for the next iteration. When the number of similar flags is greater than dissimilar flags, the source case with the highest similarity value will be selected and its associated similar cases will be compared in the next iteration. While the evaluation result indicates that the number of dissimilar flags is more, then the source case with the lowest similarity value will be chosen. As a consequence, the associated dissimilar cases will be compared in the next iteration.
- Rule 5: Occurrence of highly similar cases. When the similarity measurement of one case reaches above 75% (Rule 3), the similar association of this highly similar case will be mandatorily chosen for comparison in the next iteration, even when the rest two cases are considered dissimilar to the target case.
- Rule 6: Determination of selecting previous cases. It happens that all three cases in a single iteration have been previously compared because a past case can be associated under the one-to-many manner. For example, in Figure 9, “case 547” is associated with “9” and “956”. Under this circumstance, the compared case for the next iteration will be selected from the previous round. Based on Rule 4 and 5, the associated cases of the second most similar (or dissimilar) case are chosen for comparison.

**Figure 11:**
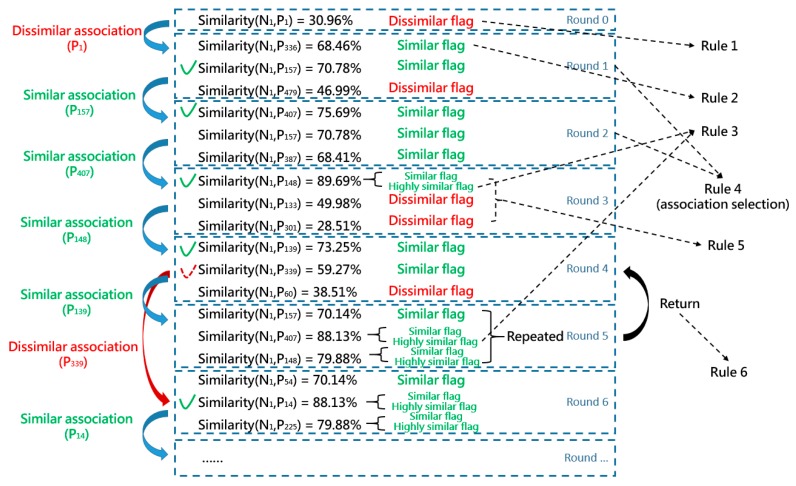
Examples of complying with Rules 1–6.

An example of Rules 1–6 is presented in Figure 11.

In Figure 11, N_1_ denotes the new case 1, while P_i_ denotes the ith past case. At first, the entry point of N_1_ was P_1_. The similarity of these two cases was 30.96%. According to Rule 1, past case 1 was considered dissimilar and assigned with a dissimilar flag. Thus, the dissimilar association of past case 1 was compared next. In round 1, the similarity of past cases 336, 157, and 479 (all of these three cases were included in the dissimilar association of past case 1) was measured and they achieved 68.46%, 70.78%, and 46.99% respectively. Based on the similarity measurement, similar flags were assigned to N_336_ and N_157_ (Rule 2), while N_479_ was marked with a dissimilar flag (Rule 1). The similar association of N_157_ was selected for comparison in round 2 due to Rule 4. In round 3, it complied with Rule 1, 2, 3, and 5. Similar and dissimilar flags were assigned to each case according to their similarity measurements (Rule 1 and 2). A highly similar flag was additionally assigned to past case 148 (Rule 3). Though the number of dissimilar flags was greater than similar flags, the similarity measurement of N_407_ was up to 75.69%, indicating that N_407_ was considered highly similar to N_1_. Therefore, the similar association of N_407_ was mandatorily selected for comparison in round 4 (Rule 5). In round 5, unfortunately, all associated cases of past case 139 were previously compared. Thus, past case 339 from round 4 was then selected for comparison in round 6 according to Rule 4 and 6. The similarity comparison continued until the termination condition was met.

In regards to the termination condition, it is defined that the maximum iteration number achieves at 100. Under the worst circumstance, 300 past cases are compared during the process of case retrieval. However, this situation is highly impossible because similar cases are associated with each other and they will repeatedly appear during the iterations. A past case may be associated with several ones. For example, in Figure 9, “case 547” was associated with “9” and “956”. Thus, the similarity of the new case and case “547” was only measured once and then recorded. The output of case retrieval is the most similar past case and it is treated as inputs to the next step of case-based reasoning (Reuse).

## 5. Experiments and Discussions

For verifying the effectiveness of our proposal, we conducted extensive experiments by retrieving agricultural cases from a case base. Totally, 500 past cases were stored in this case base and 150 new cases were prepared for testing purpose. All the cases were structured, following the proposed associated case representation “problem–solution-association”. Currently, we adopted simulated data, which were generated within a given range. As this associated case representation was developed within a European research project, named Aggregate Farming in the Cloud (AFarCloud), we were expecting to receive data from real fields as soon as the sensor deployment was finished. The data we were using could be found in the following link: https://github.com/ZhaoyuZHAI/Case-base/tree/master.

In this paper, agricultural cases focused on pest management problems. According to the conclusion in Section 3 and discussions with partners from the project, environmental and crop/plant related data were considered in the problem description part of agricultural cases, including pest quantity, pest stage, infected area, crop growth stage, crop planting density, the maximum and minimum temperature during the day, humidity, prediction of rainfall, sunlight, and wind speed. It was assumed that all the information was complete and no missing attributes existed in the dataset.

The associated case representation method was compared with the typical attribute-value pair representation in case retrieval tasks. The comparison considered two criteria: accuracy and efficiency. On the one hand, the accuracy of case retrieval specified that the retrieved past case should have great commonalities with the target one. On the other hand, the efficiency of case retrieval was determined by the number of visited cases. Visiting fewer cases means a higher efficiency.

In the experiment, an entry point was defined as the first past case, which was compared with the target case. Following the workflow in Figure 10, the entry point was randomly selected from the case base. However, we were verifying every possibility of case combinations for the start. Thus, the experiment was conducted 75,000 (150 × 500) times.

The accuracy considers the average precision [56] of retrieved top three similar cases. The formula of the average precision is defined in Equation (1).

(1) $$\mathrm{Average}\;\mathrm{precision}=\;\frac{\mathrm{TP}}{\mathrm{TP}+\mathrm{FP}}\;\left(\%\right)$$

where TP means true positive and FP stands for false positive.

**Figure 12:**
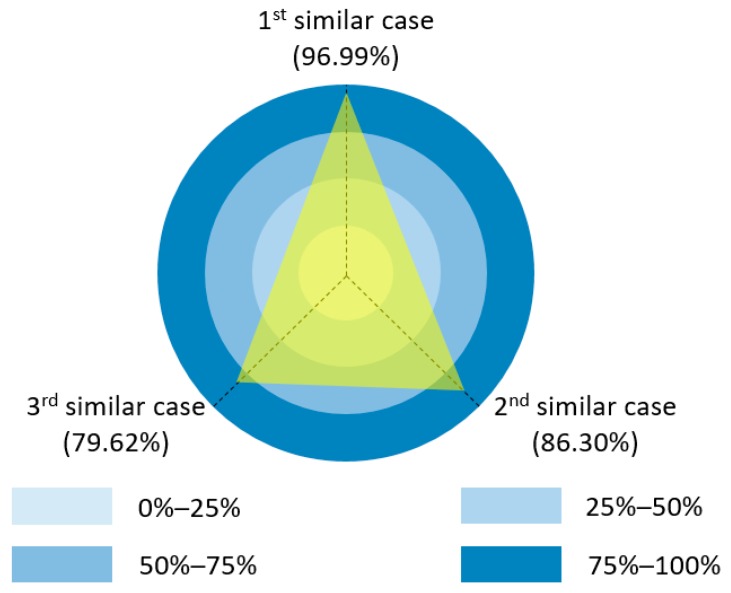
Average precision of retrieved top three similar cases.

The result of average precision is shown in Figure 12.

In Figure 12, the accuracy of retrieved top three similar cases achieved at 96.99% (72,742/75,000), 86.30% (64,725/75,000), and 79.62% (59,715/75,000) respectively. The experiment result demonstrated that the proposed associated case representation method enabled fast case retrieval with a great accuracy in most situations. Under those circumstances that failed to identify the most similar case, the second similar case usually took the top position because the first similar case was not visited during the process of case retrieval.

**Figure 13:**
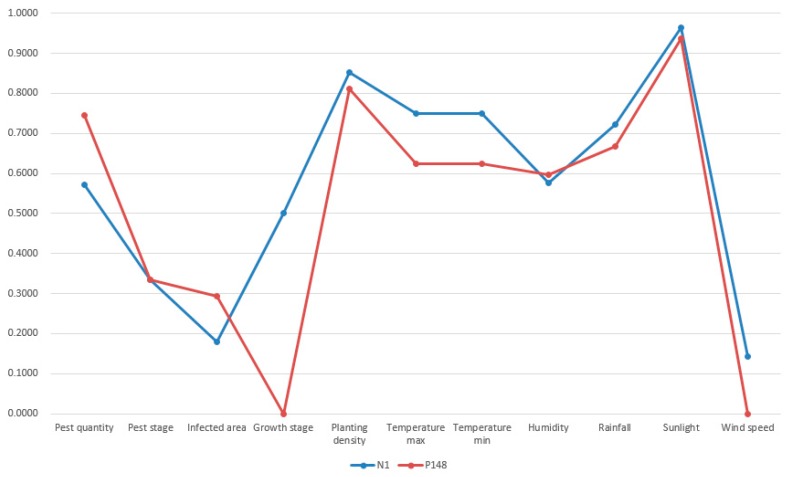
Visualization of the new case 1 and past case 148.

**Figure 14:**
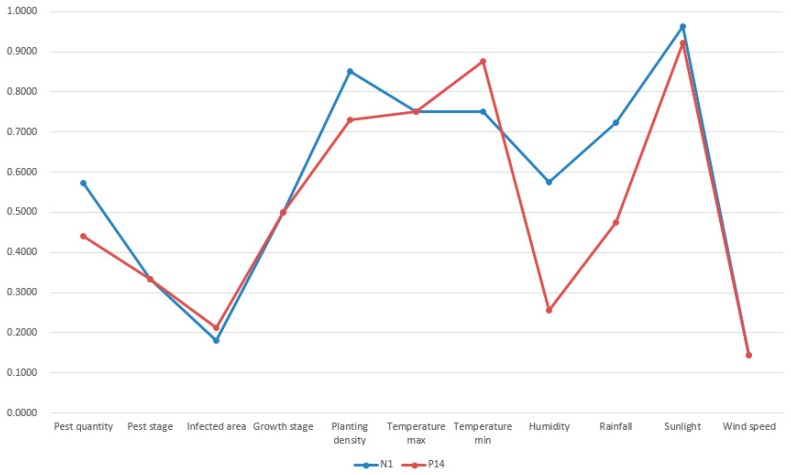
Visualization of the new case 1 and past case 14.

**Figure 15:**
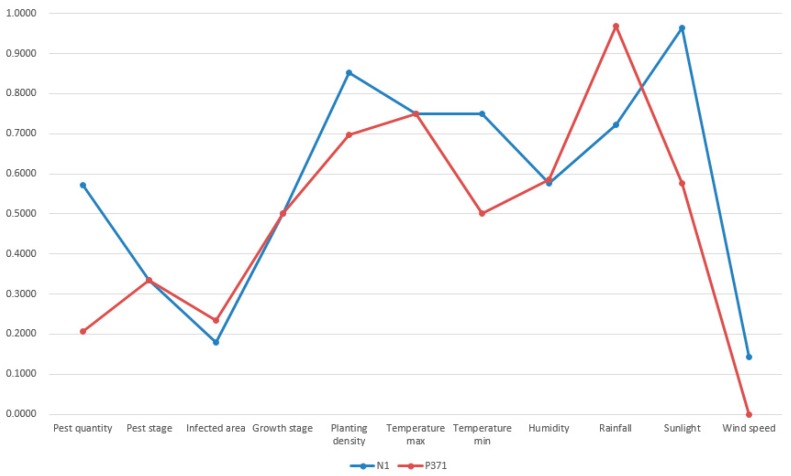
Visualization of the new case and past case 371.

**Figure 16:**
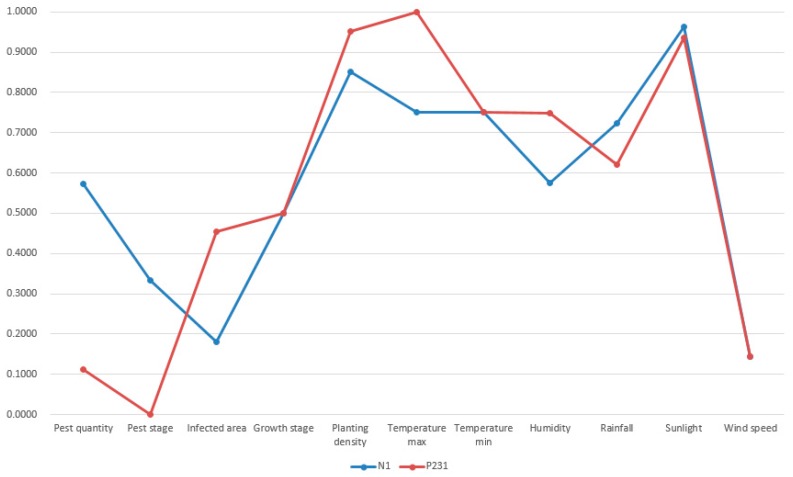
Visualization of the new case 1 and past case 231.

As we specified in Section 4.2, the termination condition was set as the iteration number reaches 100. Thus, the first most similar case was possibly missed under few circumstances. However, this was acceptable because case-based reasoning did not necessarily require the successful retrieval of the most similar case. No matter the first most similar or the second most similar past cases, they are both not exactly the same as the target case [57]. Apart from case retrieval, the rest of processes in case-based reasoning like revision can adapt the solution of the retrieved past case to the new situation. Thus, the successful retrieval of a relative similar past case is enough. A visualization of the new case (N_1_), retrieved top four past cases (P_148_, P_14_, P_371_, and P_231_) is shown in Figure 13, Figure 14, Figure 15 and Figure 16.

In Figure 13, Figure 14, Figure 15 and Figure 16, the target case was new case 1 and the entry point was past case 1. The retrieval result was past case 14, 371, and 231 with the similarity measurement of 88.13%, 88.02%, and 87.11% respectively. The most similar case in the case base was actually past case 148 with the similarity measurement of 89.08%. Though past case 148 was associated with other cases, it was not visited due to the limitation of iterations and association constructions. The normalized attributes of these five cases are presented in Table 3.

For evaluating the differences between the new case and retrieved past cases, the data deviation (DD) function was adopted [58] here. Generally, a smaller value of data deviation indicates that two compared cases were similar. The result of data deviation is shown in Table 4.

From the result in Figure 13, Figure 14, Figure 15 and Figure 16 and Table 4, it was concluded that all retrieved past cases were similar to the target one. Meanwhile, there were minor differences between retrieved top four past cases, compared with the target one. The trend lines of these past cases all greatly matched with the target one. Thus, the accuracy of fast case retrieval with the associated case representation method was proved.

**Figure 17:**
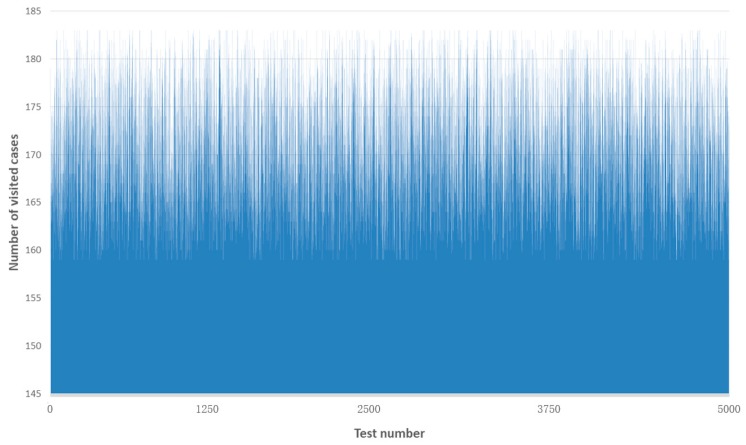
Result of the number of visited cases.

In regards to the efficiency, we evaluated the retrieval efficiency when adopting the associated case representation method. The retrieval efficiency was determined by the number of visited cases. Traditionally, all cases have to be visited when using the typical attribute-value pair representation. As a consequence, the number of visited cases was 500. Differing from the typical approach, fast case retrieval with the associated case representation measures the similarity of associated cases preferentially. The result of the number of visited cases is presented in Figure 17, indicating the retrieval efficiency of each test. The fewer the number of visited cases, the greater efficiency of case retrieval will be.

In Figure 17, we selected the result of the first 5000 tests for visualization (new cases 1–10 with entry points of past cases 1–500 respectively). For retrieving the similar past case, the minimum number of visited cases was 159 while the maximum number of visited cases was 183. The average number of visited cases for these 5000 tests was approximately 170 (169.7) cases. In regards to the whole 75,000 tests, the average number of visited cases was around 169 (168.6) cases. Thus, compared with the typical case retrieval approach (500 visited cases), it was proved that our proposal could retrieve similar past cases by visiting a fewer number of cases in the case base. Moreover, it was concluded that the number of visited cases varied in the entry point of the fast case retrieval algorithm.

In conclusion, the associated case representation method proposed in this paper enabled fast case retrieval with great accuracy and efficiency.

## 6. Conclusions and Future Work

This paper focused on proposing an associated case representation method for defining agricultural cases in a case-based reasoning system. Traditional case representation methods may lead to inefficient case retrieval when a large volume of cases is stored in the case base, because they ignore the association relations between cases. Thus, an associated case representation method was presented in this paper, following a “problem–solution-association” structure. Differing from the typical attribute-value pair representation, the novelty of our proposal is that it investigates the interconnection between cases. The relation not only considers similar associations, but also those cases that are dissimilar to the target one. On the one hand, similar associations enable fast case retrieval because similar cases are selected for comparison preferentially. On the other hand, dissimilar associations are helpful to match a relative similar case at the very beginning of case retrieval. The experimental result demonstrates that the proposed case representation method enables fast case retrieval by visiting a fewer number of cases and it guarantees the retrieval accuracy at the same time. Case retrieval plays a key role in case-based reasoning and the rest of processes cannot further proceed without successful case retrieval. Thus, the proposed associated case representation method has great potential in any case-based reasoning systems. Intelligent agricultural systems can benefit from adopting the case-based reasoning technique and the proposed associated case representation method for providing farmers with decision supports about managing agricultural activities. Moreover, our proposal offers a chance of managing agricultural knowledge under an efficient manner.

This work was developed within the AFarCloud project. In regards to future work, we are expecting to receive data from real fields for verifying the proposed associated case representation method and the fast retrieval approach. Furthermore, according to the experiment result, we are aware that the adopted fast case retrieval algorithm lacks consideration over mining the dissimilar association relations between cases. Thus, this algorithm should be further improved. Lastly, we are going to verify our proposal with a dataset that contains more volumes of data.

## Figures and Tables

**Table 1:** Key features of environmental data in agricultural cases.

Feature Name	Content
Soil	Soil type, Soil condition, location, area, etc.
Temperature	The minimum and maximum temperature value.
Humidity	Humidity value
Sunlight	Sunlight value and radiation time.
Wind	Wind speed and direction
Pests	Pest type, quantity, occurrence area, severity, etc.
Diseases	Disease name, disease stage, occurrence area, severity, etc.

**Table 2:** Key features of crop/plant related data in agricultural cases.

Feature Name	Content
Crop	Crop type, growth stage, yield, stress, dry weight, etc.
Planting	Area and planting density

**Table 3:** Normalized attributes of new case 1, past case 148, 14, 371, and 231.

Case ID	New Case 1	Past Case 148	Past Case 14	Past Case 371	Past Case 231
Pest quantity	0.5718	0.7443	0.4397	0.2064	0.1121
Pest stage	0.3333	0.3333	0.3333	0.3333	0.0000
Infected area	0.1800	0.2933	0.2133	0.2333	0.4533
Growth stage	0.5000	0.0000	0.5000	0.5000	0.5000
Planting density	0.8517	0.8110	0.7297	0.6977	0.9506
Temperature max	0.7500	0.6250	0.7500	0.7500	1.0000
Temperature min	0.7500	0.6250	0.8750	0.5000	0.7500
Humidity	0.5752	0.5968	0.2556	0.5860	0.7476
Rainfall	0.7221	0.6681	0.4751	0.9692	0.6207
Sunlight	0.9637	0.9354	0.9214	0.5759	0.9354
Wind speed	0.1429	0.0000	0.1429	0.0000	0.1429

**Table 4:** The result of data deviation between the new case and retrieved past cases.

	DD (N_1_,P_148_)	DD (N_1_,P_14_)	DD (N_1_,P_371_)	DD (N_1_,P_231_)
Value	0.2539	0.2588	0.2593	0.3002
